# Supplementary material for: PEAR, a flexible fluorescent reporter for the identification and enrichment of successfully prime edited cells
Source: eLife. 2022 Feb 23;11:e69504. doi: 10.7554/eLife.69504 (PMC8865850; doi:10.7554/eLife.69504)
Supplement: Supplementary file 1. — Forward and reverse PCR primers, PCR product sizes, and a detailed PCR protocol suitable for detecting integrated PEAR plasmids are provided. [file elife-69504-supp1.docx]

**Supplementary File 1 – Primers and PCR condition to detect PEAR plasmid integration**

| **Primer name** | **Primer sequence** |  |  |
| --- | --- | --- | --- |
|  |  |  |  |
| PEAR-detect-for | GCTGACTTAAAGGGGACCAACACAT |  |  |
| PEAR-detect-rev | TTAGTGAACCGTCAGATCCGC |  |  |
|  |  |  |  |
| **PEAR plasmid** | **PCR product size (bp)** |  |  |
|  |  |  |  |
| PEAR-GFP | 443 |  |  |
| PEAR-mScarlet | 488 |  |  |
|  |  |  |  |
| **PCR mix** |  |  |  |
|  |  |  |  |
| gDNA | 150 ng |  |  |
| Q5 polymerase | 0.25 µL |  |  |
| 10 µM primers | 1.25 – 1.25 µL |  |  |
| 10 mM dNTPs | 0.5 µL |  |  |
| 5x Q5 reaction buffer | 5 µL |  |  |
| DNase free water | to 25 µL |  |  |
|  |  |  |  |
| **PCR protocol** |  |  |  |
|  | T [°C] | t [s] |  |
| Initial denaturation | 98 | 30 |  |
| Denaturation | 98 | 10 | 35x |
| Annealing | 68 | 15 |  |
| Extension | 72 | 15 |  |
| Final extension | 72 | 120 |  |
| Hold | to 4 | ∞ |  |
