# Supplementary material for: PEAR, a flexible fluorescent reporter for the identification and enrichment of successfully prime edited cells
Source: eLife. 2022 Feb 23;11:e69504. doi: 10.7554/eLife.69504 (PMC8865850; doi:10.7554/eLife.69504)
Supplement: Supplementary file 2. — This file contains all of the plasmids used in the study with references and additional comments. [file elife-69504-supp2.docx]

**Supplementary File 2 – List of plasmids used in this study**

| **Plasmid name** | **Reference** | **Comment** |
| --- | --- | --- |
|  |  |  |
| **General** |  |  |
| pCMV-PE2 | Anzalone et al. | Addgene #132775 |
| pX330-Flag-wtSpCas9-H840A | Unpublished | Welker Lab, Addgene #80453 |
| pX330-Flag-dSpCas9 | Kulcsar et al. | Addgene #92113 |
|  | | |
| **PEAR target plasmids** | | |
| pDAS12124_PEAR-GFP-1-preedited | This study |  |
| pDAS12125_PEAR-GFP-1 | This study |  |
| PEAR-GFP-2 | Tálas et al. | pAT9651-BEAR-GFP (Addgene #162989) |
| PEAR-mScaret | Tálas et al. | pAT9752-BEAR-mScarlet (Addgene #162991) |
| pAT9624-BEAR-cloning | Tálas et al. | Addgene #162986 |
| 12420_PEAR-GFP_insGT-AAGT | This study |  |
| 12421_PEAR-GFP_insG-GT | This study |  |
| 12422_PEAR-GFP_insCAG_subGT | This study |  |
| 12423_PEAR-GFP_insGT_subAAGT | This study |  |
| 12424_PEAR-GFP_insAG_subGT | This study |  |
| 12425_PEAR-GFP_del_CAGA_to_CAG | This study |  |
| 12426_PEAR-GFP_del_CAGAA_to_CAG | This study |  |
| 12427_PEAR-GFP_del_CTAG_to_CAG | This study |  |
| 12428_PEAR-GFP_del_GAT_to_GT | This study |  |
| 12429_PEAR-GFP_del_GACT_to_GT | This study |  |
| 12430_PEAR-GFP_del_CACAG_to_CAG | This study |  |
| 12431_PEAR-GFP_delTGA | This study |  |
| 12432_PEAR-GFP_CAG-CT-AAGT | This study |  |
| 12433_PEAR-GFP_CAG-AC-TGCG | This study |  |
| 12434_PEAR-GFP_CAG-CT-TGCG | This study |  |
| 12435_PEAR-GFP_CAA-AC-AAGT | This study |  |
| 12436_PEAR-GFP_CAA-CT-AAGT | This study |  |
| 12439_PEAR-GFP_CAG-AC | This study |  |
| 12440_PEAR-GFP_CAG-CT | This study |  |
| 12443_PEAR-GFP_CAG-GT-AAGT | This study |  |
|  |  |  |
| **2in1 PEAR plasmids** | | |
| pDAS12342_PEAR-GFP_2in1 | This study |  |
| 12488_2in1_EGFPs7_9 PBS 24 RT | This study |  |
| 12489_2in1_EGFPs7_12 PBS 24 RT | This study |  |
| 12490_2in1_EGFPs7_15 PBS 24 RT | This study |  |
| 12488_2in1_EGFPs2_9 PBS 24 RT | This study |  |
| 12489_2in1_EGFPs2_12 PBS 24 RT | This study |  |
| 12490_2in1_EGFPs2_15 PBS 24 RT | This study |  |
|  |  |  |
| **pegRNA plasmids** | | |
| 12069-U6-pegRNA-mCherry | This study | target cloning plasmid |
| 12222-U6-pegRNA-BFP | This study | target cloning plasmid |
|  |  |  |
| 12227_U6-pegRNA-PEAR-GFP_10PBS-16RT_mCherry | This study | targeting the PEAR-GFP plasmid |
| 12228_U6-pegRNA-PEAR-GFP_13PBS-16RT_mCherry | This study | targeting the PEAR-GFP plasmid |
| 12229_U6-pegRNA-PEAR-GFP_16PBS-16RT_mCherry | This study | targeting the PEAR-GFP plasmid |
| 12230_U6-pegRNA-PEAR-GFP_10PBS-24RT_mCherry | This study | targeting the PEAR-GFP plasmid |
| 12231_U6-pegRNA-PEAR-GFP_13PBS-24RT_mCherry | This study | targeting the PEAR-GFP plasmid |
| 12232_U6-pegRNA-PEAR-GFP_16PBS-24RT_mCherry | This study | targeting the PEAR-GFP plasmid |
| 12233_U6-pegRNA-PEAR-GFP_10PBS-33RT_mCherry | This study | targeting the PEAR-GFP plasmid |
| 12234_U6-pegRNA-PEAR-GFP_13PBS-33RT_mCherry | This study | targeting the PEAR-GFP plasmid |
| 12235_U6-pegRNA-PEAR-GFP_16PBS-33RT_mCherry | This study | targeting the PEAR-GFP plasmid |
|  |  |  |
| 12248_U6-pegRNA-PEAR-GFP-2_10PBS-22RT_mCherry | This study | targeting the PEAR-GFP-2 plasmid and the PEAR-GFP cell line |
| 12249_U6-pegRNA-PEAR-GFP-2_13PBS-22RT_mCherry | This study | targeting the PEAR-GFP-2 plasmid and the PEAR-GFP cell line |
| 12250_U6-pegRNA-PEAR-GFP-2_16PBS-22RT_mCherry | This study | targeting the PEAR-GFP-2 plasmid and the PEAR-GFP cell line |
| 12251_U6-pegRNA-PEAR-GFP-2_10PBS-26RT_mCherry | This study | targeting the PEAR-GFP-2 plasmid and the PEAR-GFP cell line |
| 12252_U6-pegRNA-PEAR-GFP-2_13PBS-26RT_mCherry | This study | targeting the PEAR-GFP-2 plasmid and the PEAR-GFP cell line |
| 12253_U6-pegRNA-PEAR-GFP-2_16PBS-26RT_mCherry | This study | targeting the PEAR-GFP-2 plasmid and the PEAR-GFP cell line |
| 12254_U6-pegRNA-PEAR-GFP-2_10PBS-33RT_mCherry | This study | targeting the PEAR-GFP-2 plasmid and the PEAR-GFP cell line |
| 12255_U6-pegRNA-PEAR-GFP-2_13PBS-33RT_mCherry | This study | targeting the PEAR-GFP-2 plasmid and the PEAR-GFP cell line |
| 12256_U6-pegRNA-PEAR-GFP-2_16PBS-33RT_mCherry | This study | targeting the PEAR-GFP-2 plasmid and the PEAR-GFP cell line |
|  |  |  |
| 12257_U6-pegRNA-PEAR-mScarlet-target1_10PBS-31RT_BFP | This study | targeting the PEAR-mScarlet - target1 plasmid and the PEAR-mScarlet cell line |
| 12258_U6-pegRNA-PEAR-mScarlet-target1_13PBS-31RT_BFP | This study | targeting the PEAR-mScarlet - target1 plasmid and the PEAR-mScarlet cell line |
| 12259_U6-pegRNA-PEAR-mScarlet-target1_16PBS-31RT_BFP | This study | targeting the PEAR-mScarlet - target1 plasmid and the PEAR-mScarlet cell line |
| 12260_U6-pegRNA-PEAR-mScarlet-target1_10PBS-35RT_BFP | This study | targeting the PEAR-mScarlet - target1 plasmid and the PEAR-mScarlet cell line |
| 12261_U6-pegRNA-PEAR-mScarlet-target1_13PBS-35RT_BFP | This study | targeting the PEAR-mScarlet - target1 plasmid and the PEAR-mScarlet cell line |
| 12262_U6-pegRNA-PEAR-mScarlet-target1_16PBS-35RT_BFP | This study | targeting the PEAR-mScarlet - target1 plasmid and the PEAR-mScarlet cell line |
| 12263_U6-pegRNA-PEAR-mScarlet-target1_10PBS-39RT_BFP | This study | targeting the PEAR-mScarlet - target1 plasmid and the PEAR-mScarlet cell line |
| 12264_U6-pegRNA-PEAR-mScarlet-target1_13PBS-39RT_BFP | This study | targeting the PEAR-mScarlet - target1 plasmid and the PEAR-mScarlet cell line |
| 12265_U6-pegRNA-PEAR-mScarlet-target1_16PBS-39RT_BFP | This study | targeting the PEAR-mScarlet - target1 plasmid and the PEAR-mScarlet cell line |
|  |  |  |
| 12266_U6-pegRNA-PEAR-mScarlet-target2_10PBS-38RT_BFP | This study | targeting the PEAR-mScarlet - target2 |
| 12267_U6-pegRNA-PEAR-mScarlet-target2_13PBS-38RT_BFP | This study | targeting the PEAR-mScarlet - target2 |
| 12268_U6-pegRNA-PEAR-mScarlet-target2_16PBS-38RT_BFP | This study | targeting the PEAR-mScarlet - target2 |
| 12269_U6-pegRNA-PEAR-mScarlet-target2_10PBS-42RT_BFP | This study | targeting the PEAR-mScarlet - target2 |
| 12270_U6-pegRNA-PEAR-mScarlet-target2_13PBS-42RT_BFP | This study | targeting the PEAR-mScarlet - target2 |
| 12271_U6-pegRNA-PEAR-mScarlet-target2_16PBS-42RT_BFP | This study | targeting the PEAR-mScarlet - target2 |
| 12272_U6-pegRNA-PEAR-mScarlet-target2_10PBS-46RT_BFP | This study | targeting the PEAR-mScarlet - target2 |
| 12273_U6-pegRNA-PEAR-mScarlet-target2_13PBS-46RT_BFP | This study | targeting the PEAR-mScarlet - target2 |
| 12274_U6-pegRNA-PEAR-mScarlet-target2_16PBS-46RT_BFP | This study | targeting the PEAR-mScarlet - target2 |
|  |  |  |
| 12410_EMX1 pegRNA_15 PBS 13 RT_TagBFP | This study | Targeting EMX1 genomic target |
| 12411_RNF2 pegRNA_15 PBS 14 RT_TagBFP | This study | Targeting RNF2 genomic target |
| 12412_FANCF pegRNA_8 PBS 17 RT_TagBFP | This study | Targeting FANCF genomic target |
| 12413_HEK3 pegRNA13 PBS 10 RT_TagBFP | This study | Targeting HEK3 genomic target |
| 12414_HEK4 pegRNA_8 PBS 10 RT_TagBFP | This study | Targeting HEK4 genomic target |
| 12454_HEK3 +1 CTT ins pegRNA_BFP | This study | Targeting HEK3 genomic target |
| 12455_EMX1 +5G del pegRNA_BFP | This study | Targeting EMX1 genomic target |
| 12456_RUNX1 +1 ATG ins pegRNA_BFP | This study | Targeting RUNX1 genomic target |
| 12457_RUNX1 +2 G del pegRNA_BFP | This study | Targeting RUNX1 genomic target |
| 12460_DNMT +3-5 AAG del pegRNA_BFP | This study | Targeting DNMT genomic target |
| 12462_FANCF +2C del_+5GtoT pegRNA_BFP | This study | Targeting FANCF genomic target |
| 12474_HBB_+4 A to T_pegRNA_BFP | This study | Targeting HBB genomic target |
| 12475_HEXA_+1 TATC ins_pegRNA_BFP | This study | Targeting HEXA genomic target |
| 12476_PRNP_+6 G to T_pegRNA_BFP | This study | Targeting PRNP genomic target |
|  |  |  |
| 12449_PEAR-GFP pegRNA_G-GT-TGCG_10 PBS 24 RT |  |  |
| 12450_PEAR-GFP pegRNA_A-GT-AAGT_10 PBS 24 RT |  |  |
| 12452_PEAR-GFP pegRNA_G-GT_10 PBS 24 RT |  |  |
|  |  |  |
| **sgRNA plasmids** | |  |
| 12136_U6-sgRNA-PEAR-GFP_nick(+89)_mCherry | This study | Secondary nick on the PEAR-GFP plasmid |
| 12137_U6-sgRNA-PEAR-GFP_nick(+17)_mCherry | This study | Secondary nick on the PEAR-GFP plasmid |
| 12138_U6-sgRNA-PEAR-GFP_nick(-59)_mCherry | This study | Secondary nick on the PEAR-GFP plasmid |
| 12139_U6-sgRNA-PEAR-GFP_nick(-100)_mCherry | This study | Secondary nick on the PEAR-GFP plasmid |
| 12140_U6-sgRNA-PEAR-GFP_nick(-162)_mCherry | This study | Secondary nick on the PEAR-GFP plasmid |
|  |  |  |
| 12198_U6-sgRNA-PEAR-GFP-2_nick(+103)_mCherry | This study | Secondary nick on the PEAR-GFP-2 plasmid, and in the PEAR-GFP cell line |
| 12199_U6-sgRNA-PEAR-GFP-2_nick(+126)_mCherry | This study | Secondary nick on the PEAR-GFP-2 plasmid, and in the PEAR-GFP cell line |
| 12200_U6-sgRNA-PEAR-GFP-2_nick(+ 17)_mCherry | This study | Secondary nick on the PEAR-GFP-2 plasmid, and in the PEAR-GFP cell line |
|  |  |  |
| 12210_U6-sgRNA-PEAR-mScarlet_nick(+103)_BFP | This study | Secondary nick on the PEAR-mScarlet plasmid and in the PEAR-mScarlet cell line |
| 12211_U6-sgRNA-PEAR-mScarlet_nick(+126)_BFP | This study | Secondary nick on the PEAR-mScarlet plasmid and in the PEAR-mScarlet cell line |
| 12212_U6-sgRNA-PEAR-mScarlet_nick(+ 17)_BFP | This study | Secondary nick on the PEAR-mScarlet plasmid and in the PEAR-mScarlet cell line |
|  |  |  |
| 12415_U6-sgRNA-EMX1-nick(-57)_BFP | This study | Secondary nick in the genome |
| 12416_U6-sgRNA-RNF2 +41 nick_BFP | This study | Secondary nick in the genome |
| 12417_U6-sgRNA-FANCF +48 nick_BFP | This study | Secondary nick in the genome |
| 12418_U6-sgRNA-HEK3 +90 nick_BFP | This study | Secondary nick in the genome |
| 12419_U6-sgRNA-HEK4 -95 nick_BFP | This study | Secondary nick in the genome |
| 12463_U6-sgRNA-EMX1 +53 nick_BFP | This study | Secondary nick in the genome |
| 12464_U6-sgRNA-RUNX1 +38 nick_BFP | This study | Secondary nick in the genome |
| 12466_U6-sgRNA-DNMT +49 nick_BFP | This study | Secondary nick in the genome |
| 12479_U6-sgRNA-HBB nick_BFP | This study | Secondary nick in the genome |
| 12480_U6-sgRNA-HEXA nick_BFP | This study | Secondary nick in the genome |
| 12481_U6-sgRNA-PRNP nick_BFP | This study | Secondary nick in the genome |
|  |  |  |
| pAT9922_U6-sgRNA-mock-mCherry | This study | mock sgRNA used in when not nicking the plasmid/genome |
| 9762_U6-sgRNA-mock-TagBFP | This study | mock sgRNA used in when not nicking the plasmid/genome |
