# Supplementary material for: PEAR, a flexible fluorescent reporter for the identification and enrichment of successfully prime edited cells
Source: eLife. 2022 Feb 23;11:e69504. doi: 10.7554/eLife.69504 (PMC8865850; doi:10.7554/eLife.69504)
Supplement: Supplementary file 3. — This file contains the oligonucleotide sequences used for molecular cloning or PCR. [file elife-69504-supp3.docx]

**Supplementary File 3 – List of oligonucleotides used in this study**

| **Oligonucleotide name** | **Oligonucleotide sequence** |
| --- | --- |
| 12124_L1 | CGTGCAGGTAAGTCCTAGACTGCGGGTTTGACATTC |
| 12124_L2 | AAATGAATGTCAAACCCGCAGTCTAGGACTTACCTG |
| 12125_L1 | CGTGCAGACAAGTCCTAGACTGCGGGTTTGACATTC |
| 12125_L2 | AAATGAATGTCAAACCCGCAGTCTAGGACTTGTCTG |
| 12227-12235-spacer-L1 | CACCGAATGTCAAACCCGCAGTCT |
| 12227-12235-spacer-L2 | AAACAGACTGCGGGTTTGACATTC |
| 12227_16RT_10PBS_for | GTGCGCAGGTAAGTCCTAGACTGCGGGTTT |
| 12227_16RT_10PBS_rev | AAAATCTAGGACTTACCTGCAAACCCGCAG |
| 12228_16RT_13PBS_for | GTGCGCAGGTAAGTCCTAGACTGCGGGTTTGAC |
| 12228_16RT_13PBS_rev | AAAATCTAGGACTTACCTGCGTCAAACCCGCAG |
| 12229_16RT_16PBS_for | GTGCGCAGGTAAGTCCTAGACTGCGGGTTTGACATT |
| 12229_16RT_16PBS_rev | AAAATCTAGGACTTACCTGCAATGTCAAACCCGCAG |
| 12230_24RT_10PBS_for | GTGCGGCTACGTGCAGGTAAGTCCTAGACTGCGGGTTT |
| 12230_24RT_10PBS_rev | AAAATCTAGGACTTACCTGCACGTAGCCAAACCCGCAG |
| 12231_24RT_13PBS_for | GTGCGGCTACGTGCAGGTAAGTCCTAGACTGCGGGTTTGAC |
| 12231_24RT_13PBS_rev | AAAATCTAGGACTTACCTGCACGTAGCCGTCAAACCCGCAG |
| 12232_24RT_16PBS_for | GTGCGGCTACGTGCAGGTAAGTCCTAGACTGCGGGTTTGACATT |
| 12232_24RT_16PBS_rev | AAAATCTAGGACTTACCTGCACGTAGCCAATGTCAAACCCGCAG |
| 12233_33RT_10PBS_for | GTGCATGCCCGAAGGCTACGTGCAGGTAAGTCCTAGACTGCGGGTTT |
| 12233_33RT_10PBS_rev | AAAATCTAGGACTTACCTGCACGTAGCCTTCGGGCATAAACCCGCAG |
| 12234_33RT_13PBS_for | GTGCATGCCCGAAGGCTACGTGCAGGTAAGTCCTAGACTGCGGGTTTGAC |
| 12234_33RT_13PBS_rev | AAAATCTAGGACTTACCTGCACGTAGCCTTCGGGCATGTCAAACCCGCAG |
| 12235_33RT_16PBS_for | GTGCATGCCCGAAGGCTACGTGCAGGTAAGTCCTAGACTGCGGGTTTGACATT |
| 12235_33RT_16PBS_rev | AAAATCTAGGACTTACCTGCACGTAGCCTTCGGGCATAATGTCAAACCCGCAG |
| 12248-12256-spacer-L1 | CACCGTTCAAGTCCGCCATGCCCGA |
| 12248-12256-spacer-L2 | AAACTCGGGCATGGCGGACTTGAAC |
| 12248_peg1 | GTGCACTTACCTGCACGTAGCCTTCGTCAAGTCCGC |
| 12248_peg2 | AAAAGCGGACTTGACGAAGGCTACGTGCAGGTAAGT |
| 12249_peg1 | GTGCACTTACCTGCACGTAGCCTTCGTCAAGTCCGCCAT |
| 12249_peg2 | AAAAATGGCGGACTTGACGAAGGCTACGTGCAGGTAAGT |
| 12250_peg1 | GTGCACTTACCTGCACGTAGCCTTCGTCAAGTCCGCCATGCC |
| 12250_peg2 | AAAAGGCATGGCGGACTTGACGAAGGCTACGTGCAGGTAAGT |
| 12251_peg1 | GTGCATGCACTTACCTGCACGTAGCCTTCGTCAAGTCCGC |
| 12251_peg2 | AAAAGCGGACTTGACGAAGGCTACGTGCAGGTAAGTGCAT |
| 12252_peg1 | GTGCATGCACTTACCTGCACGTAGCCTTCGTCAAGTCCGCCAT |
| 12252_peg2 | AAAAATGGCGGACTTGACGAAGGCTACGTGCAGGTAAGTGCAT |
| 12253_peg1 | GTGCATGCACTTACCTGCACGTAGCCTTCGTCAAGTCCGCCATGCC |
| 12253_peg2 | AAAAGGCATGGCGGACTTGACGAAGGCTACGTGCAGGTAAGTGCAT |
| 12254_peg1 | GTGCGCAGTCTATGCACTTACCTGCACGTAGCCTTCGTCAAGTCCGC |
| 12254_peg2 | AAAAGCGGACTTGACGAAGGCTACGTGCAGGTAAGTGCATAGACTGC |
| 12255_peg1 | GTGCGCAGTCTATGCACTTACCTGCACGTAGCCTTCGTCAAGTCCGCCAT |
| 12255_peg2 | AAAAATGGCGGACTTGACGAAGGCTACGTGCAGGTAAGTGCATAGACTGC |
| 12256_peg1 | GTGCGCAGTCTATGCACTTACCTGCACGTAGCCTTCGTCAAGTCCGCCATGCC |
| 12256_peg2 | AAAAGGCATGGCGGACTTGACGAAGGCTACGTGCAGGTAAGTGCATAGACTGC |
| 12257-12265-spacer-L1 | CACCGTGATGAACTTCGAGGACGG |
| 12257-12265-spacer-L2 | AAACCCGTCCTCGAAGTTCATCAC |
| 12257_peg1 | GTGCACTTACCTGCGTCACGGTCACGGCGCCGCCGTCCTCGAAGT |
| 12257_peg2 | AAAAACTTCGAGGACGGCGGCGCCGTGACCGTGACGCAGGTAAGT |
| 12258_peg1 | GTGCACTTACCTGCGTCACGGTCACGGCGCCGCCGTCCTCGAAGTTCA |
| 12258_peg2 | AAAATGAACTTCGAGGACGGCGGCGCCGTGACCGTGACGCAGGTAAGT |
| 12259_peg1 | GTGCACTTACCTGCGTCACGGTCACGGCGCCGCCGTCCTCGAAGTTCATCA |
| 12259_peg2 | AAAATGATGAACTTCGAGGACGGCGGCGCCGTGACCGTGACGCAGGTAAGT |
| 12260_peg1 | GTGCATGCACTTACCTGCGTCACGGTCACGGCGCCGCCGTCCTCGAAGT |
| 12260_peg2 | AAAAACTTCGAGGACGGCGGCGCCGTGACCGTGACGCAGGTAAGTGCAT |
| 12261_peg1 | GTGCATGCACTTACCTGCGTCACGGTCACGGCGCCGCCGTCCTCGAAGTTCA |
| 12261_peg2 | AAAATGAACTTCGAGGACGGCGGCGCCGTGACCGTGACGCAGGTAAGTGCAT |
| 12262_peg1 | GTGCATGCACTTACCTGCGTCACGGTCACGGCGCCGCCGTCCTCGAAGTTCATCA |
| 12262_peg2 | AAAATGATGAACTTCGAGGACGGCGGCGCCGTGACCGTGACGCAGGTAAGTGCAT |
| 12263_peg1 | GTGCGTCTATGCACTTACCTGCGTCACGGTCACGGCGCCGCCGTCCTCGAAGT |
| 12263_peg2 | AAAAACTTCGAGGACGGCGGCGCCGTGACCGTGACGCAGGTAAGTGCATAGAC |
| 12264_peg1 | GTGCGTCTATGCACTTACCTGCGTCACGGTCACGGCGCCGCCGTCCTCGAAGTTCA |
| 12264_peg2 | AAAATGAACTTCGAGGACGGCGGCGCCGTGACCGTGACGCAGGTAAGTGCATAGAC |
| 12265_peg1 | GTGCGTCTATGCACTTACCTGCGTCACGGTCACGGCGCCGCCGTCCTCGAAGTTCATCA |
| 12265_peg2 | AAAATGATGAACTTCGAGGACGGCGGCGCCGTGACCGTGACGCAGGTAAGTGCATAGAC |
| 12266-12274-spacer-L1 | CACCGGAGCGCGTGATGAACTTCG |
| 12266-12274-spacer-L2 | AAACCGAAGTTCATCACGCGCTCC |
| 12266_peg1 | GTGCACTTACCTGCGTCACGGTCACGGCGCCGCCGTCCTCGAAGTTCATCAC |
| 12266_peg2 | AAAAGTGATGAACTTCGAGGACGGCGGCGCCGTGACCGTGACGCAGGTAAGT |
| 12267_peg1 | GTGCACTTACCTGCGTCACGGTCACGGCGCCGCCGTCCTCGAAGTTCATCACGCG |
| 12267_peg2 | AAAACGCGTGATGAACTTCGAGGACGGCGGCGCCGTGACCGTGACGCAGGTAAGT |
| 12268_peg1 | GTGCACTTACCTGCGTCACGGTCACGGCGCCGCCGTCCTCGAAGTTCATCACGCGCTC |
| 12268_peg2 | AAAAGAGCGCGTGATGAACTTCGAGGACGGCGGCGCCGTGACCGTGACGCAGGTAAGT |
| 12269_peg1 | GTGCATGCACTTACCTGCGTCACGGTCACGGCGCCGCCGTCCTCGAAGTTCATCAC |
| 12269_peg2 | AAAAGTGATGAACTTCGAGGACGGCGGCGCCGTGACCGTGACGCAGGTAAGTGCAT |
| 12270_peg1 | GTGCATGCACTTACCTGCGTCACGGTCACGGCGCCGCCGTCCTCGAAGTTCATCACGCG |
| 12270_peg2 | AAAACGCGTGATGAACTTCGAGGACGGCGGCGCCGTGACCGTGACGCAGGTAAGTGCAT |
| 12271_peg1 | GTGCATGCACTTACCTGCGTCACGGTCACGGCGCCGCCGTCCTCGAAGTTCATCACGCGCTC |
| 12271_peg2 | AAAAGAGCGCGTGATGAACTTCGAGGACGGCGGCGCCGTGACCGTGACGCAGGTAAGTGCAT |
| 12272_peg1 | GTGCGTCTATGCACTTACCTGCGTCACGGTCACGGCGCCGCCGTCCTCGAAGTTCATCAC |
| 12272_peg2 | AAAAGTGATGAACTTCGAGGACGGCGGCGCCGTGACCGTGACGCAGGTAAGTGCATAGAC |
| 12273_peg1 | GTGCGTCTATGCACTTACCTGCGTCACGGTCACGGCGCCGCCGTCCTCGAAGTTCATCACGCG |
| 12273_peg2 | AAAACGCGTGATGAACTTCGAGGACGGCGGCGCCGTGACCGTGACGCAGGTAAGTGCATAGAC |
| 12274_peg1 | GTGCGTCTATGCACTTACCTGCGTCACGGTCACGGCGCCGCCGTCCTCGAAGTTCATCACGCGCTC |
| 12274_peg2 | AAAAGAGCGCGTGATGAACTTCGAGGACGGCGGCGCCGTGACCGTGACGCAGGTAAGTGCATAGAC |
| 12410_EMX1-spacer-L1 | CACCGAGTCCGAGCAGAAGAAGAA |
| 12410_EMX1-spacer-L2 | AAACTTCTTCTTCTGCTCGGACTC |
| 12410_peg1 | GTGCATGGGAGCACTTCTTCTTCTGCTCGGAC |
| 12410_peg2 | AAAAGTCCGAGCAGAAGAAGAAGTGCTCCCAT |
| 12411_RNF2-spacer-L1 | CACCGTCATCTTAGTCATTACCTG |
| 12411_RNF2-spacer-L2 | AAACCAGGTAATGACTAAGATGAC |
| 12411_peg1 | GTGCAACGAACACCTCATGTAATGACTAAGATG |
| 12411_peg2 | AAAACATCTTAGTCATTACATGAGGTGTTCGTT |
| 12412_FANCF-spacer-L1 | CACCGGAATCCCTTCTGCAGCACC |
| 12412_FANCF-spacer-L2 | AAACGGTGCTGCAGAAGGGATTCC |
| 12412_peg1 | GTGCGGAAAAGCGATCAAGGTGCTGCAGA |
| 12412_peg2 | AAAATCTGCAGCACCTTGATCGCTTTTCC |
| 12413_HEK3-spacer-L1 | CACCGGCCCAGACTGAGCACGTGA |
| 12413_HEK3-spacer-L2 | AAACTCACGTGCTCAGTCTGGGCC |
| 12413_peg1 | GTGCTCTGCCATCTCGTGCTCAGTCTG |
| 12413_peg2 | AAAACAGACTGAGCACGAGATGGCAGA |
| 12414_HEK4-spacer-L1 | CACCGGCACTGCGGCTGGAGGTGG |
| 12414_HEK4-spacer-L2 | AAACCCACCTCCAGCCGCAGTGCC |
| 12414_peg1 | GTGCTTAACCCCAACCTCCAGC |
| 12414_peg2 | AAAAGCTGGAGGTTGGGGTTAA |
| 12136_L1 | CACCGCTCGTGACCACCCTGACCTA |
| 12136_L2 | AAACTAGGTCAGGGTGGTCACGAGC |
| 12137_L1 | CACCGTTCAAGTCCGCCATGCCCGA |
| 12137_L2 | AAACTCGGGCATGGCGGACTTGAAC |
| 12138_L1 | CACCGATATGGGAAAGTTTAAAAGA |
| 12138_L2 | AAACTCTTTTAAACTTTCCCATATC |
| 12139_L1 | CACCGCAACCACTTTAAATATGTGT |
| 12139_L2 | AAACACACATATTTAAAGTGGTTGC |
| 12140_L1 | CACCGCTAACTTAAGGAGTCCCCG |
| 12140_L2 | AAACCGGGGACTCCTTAAGTTAGC |
| 12198_L1 | CACCGGACCAACACATATTTAAAG |
| 12198_L2 | AAACCTTTAAATATGTGTTGGTCC |
| 12199_L1 | CACCGATATCTGCTTGCTGACTTAA |
| 12199_L2 | AAACTTAAGTCAGCAAGCAGATATC |
| 12200_L1 | CACCGCTATTCATCCAGTATCCAC |
| 12200_L2 | AAACGTGGATACTGGATGAATAGC |
| 12210_L1 | same as: 12198_L1 |
| 12210_L2 | same as: 12198_L2 |
| 12211_L1 | same as: 12199_L1 |
| 12211_L2 | same as: 12199_L2 |
| 12212_L1 | same as: 12200_L1 |
| 12212_L2 | same as: 12200_L2 |
| 12415_L1 | CACCGCCGTTTGTACTTTGTCCTC |
| 12415_L2 | AAACGAGGACAAAGTACAAACGGC |
| 12416_L1 | CACCGTCAACCATTAAGCAAAACAT |
| 12416_L2 | AAACATGTTTTGCTTAATGGTTGAC |
| 12417_L1 | CACCGGGGTCCCAGGTGCTGACGT |
| 12417_L2 | AAACACGTCAGCACCTGGGACCCC |
| 12418_L1 | CACCGTCAACCAGTATCCCGGTGC |
| 12418_L2 | AAACGCACCGGGATACTGGTTGAC |
| 12419_L1 | CACCGTCCCTTCCTTCCACCCAGCC |
| 12419_L2 | AAACGGCTGGGTGGAAGGAAGGGAC |
| 9922_L1 | CACCGCAGACAAGTAGGGTGGGCC |
| 9922_L2 | AAACGGCCCACCCTACTTGTCTGC |
| 9762_L1 | CACCGCAGTCAAGTGCTGGAGGTG |
| 9762_L2 | AAACCACCTCCAGCACTTGACTGC |
| 12420_L1 | CGTGCAGCcTAGACTGCGGGTTTGACATTC |
| 12421_L1 | CGTGCAAAGTCcTAGACTGCGGGTTTGACATTC |
| 12422_L1 | CGTGACAAGTCcTAGACTGCGGGTTTGACATTC |
| 12423_L1 | CGTGCAGTGAGCcTAGACTGCGGGTTTGACATTC |
| 12424_L1 | CGTGCACAAGTCcTAGACTGCGGGTTTGACATTC |
| 12425_L1 | CGTGCAGAGTAAGTCcTAGACTGCGGGTTTGACATTC |
| 12426_L1 | CGTGCAGAAGTAAGTCcTAGACTGCGGGTTTGACATTC |
| 12427_L1 | CGTGCTAGGTAAGTCcTAGACTGCGGGTTTGACATTC |
| 12428_L1 | CGTGCAGGATAAGTCcTAGACTGCGGGTTTGACATTC |
| 12429_L1 | CGTGCAGGACTAAGTCcTAGACTGCGGGTTTGACATTC |
| 12430_L1 | CGTGCACAGGTAAGTCcTAGACTGCGGGTTTGACATTC |
| 12431_L1 | CGTGCAGACAAGTCcTAGACTGCGGGTTTGACATTC |
| 12432_L1 | CGTGCAGCTAAGTCcTAGACTGCGGGTTTGACATTC |
| 12433_L1 | CGTGCAGACTGCGCcTAGACTGCGGGTTTGACATTC |
| 12434_L1 | CGTGCAGCTTGCGCcTAGACTGCGGGTTTGACATTC |
| 12435_L1 | CGTGCAAACAAGTCcTAGACTGCGGGTTTGACATTC |
| 12436_L1 | CGTGCAACTAAGTCcTAGACTGCGGGTTTGACATTC |
| 12439_L1 | CGTGCAGACCcTAGACTGCGGGTTTGACATTC |
| 12440_L1 | CGTGCAGCTCcTAGACTGCGGGTTTGACATTC |
| 12443_L1 | CGTGCAGGTAAGTCcTAGACTGCGGGTTTGACATTC |
| 12444_L1 | CGTGCAGGTTGCGCcTAGACTGCGGGTTTGACATTC |
| 12445_L1 | CGTGCAAGTAAGTCcTAGACTGCGGGTTTGACATTC |
| 12447_L1 | CGTGCAGGTCcTAGACTGCGGGTTTGACATTC |
| 12420_L2 | AAATGAATGTCAAACCCGCAGTCTAGGCTG |
| 12421_L2 | AAATGAATGTCAAACCCGCAGTCTAGGACTTTG |
| 12422_L2 | AAATGAATGTCAAACCCGCAGTCTAGGACTTGT |
| 12423_L2 | AAATGAATGTCAAACCCGCAGTCTAGGCTCACTG |
| 12424_L2 | AAATGAATGTCAAACCCGCAGTCTAGGACTTGTG |
| 12425_L2 | AAATGAATGTCAAACCCGCAGTCTAGGACTTACTCTG |
| 12426_L2 | AAATGAATGTCAAACCCGCAGTCTAGGACTTACTTCTG |
| 12427_L2 | AAATGAATGTCAAACCCGCAGTCTAGGACTTACCTAG |
| 12428_L2 | AAATGAATGTCAAACCCGCAGTCTAGGACTTATCCTG |
| 12429_L2 | AAATGAATGTCAAACCCGCAGTCTAGGACTTAGTCCTG |
| 12430_L2 | AAATGAATGTCAAACCCGCAGTCTAGGACTTACCTGTG |
| 12431_L2 | AAATGAATGTCAAACCCGCAGTCTAGGACTTGTCTG |
| 12432_L2 | AAATGAATGTCAAACCCGCAGTCTAGGACTTAGCTG |
| 12433_L2 | AAATGAATGTCAAACCCGCAGTCTAGGCGCAGTCTG |
| 12434_L2 | AAATGAATGTCAAACCCGCAGTCTAGGCGCAAGCTG |
| 12435_L2 | AAATGAATGTCAAACCCGCAGTCTAGGACTTGTTTG |
| 12436_L2 | AAATGAATGTCAAACCCGCAGTCTAGGACTTAGTTG |
| 12439_L2 | AAATGAATGTCAAACCCGCAGTCTAGGGTCTG |
| 12440_L2 | AAATGAATGTCAAACCCGCAGTCTAGGAGCTG |
| 12443_L2 | AAATGAATGTCAAACCCGCAGTCTAGGACTTACCTG |
| 12444_L2 | AAATGAATGTCAAACCCGCAGTCTAGGCGCAACCTG |
| 12445_L2 | AAATGAATGTCAAACCCGCAGTCTAGGACTTACTTG |
| 12447_L2 | AAATGAATGTCAAACCCGCAGTCTAGGACCTG |
| 12449_L1 | GTGCGGCTACGTGCAGGTTGCGCcTAGACTGCGGGTTT |
| 12450_L1 | GTGCGGCTACGTGCAAGTAAGTCcTAGACTGCGGGTTT |
| 12452_L1 | GTGCGGCTACGTGCAGGTCcTAGACTGCGGGTTT |
| 12449_L2 | AAAAAAACCCGCAGTCTAGGCGCAACCTGCACGTAGCC |
| 12450_L2 | AAAAAAACCCGCAGTCTAGGACTTACTTGCACGTAGCC |
| 12452_L2 | AAAAAAACCCGCAGTCTAGGACCTGCACGTAGCC |
| 12454_L1 | GTGCTCTGCCATCAAAGCGTGCTCAGTCTG |
| 12455_L1 | GTGCGTGATGGGAGCCTTCTTCTTCTGCTCGGA |
| 12456_L1 | GTGCTGTCTGAAGCCATCCATGCTTCCTCCTGAAAAT |
| 12457_L1 | GTGCTGTCTGAAGCCATGCTTCCTCCTGAAAAT |
| 12460_L1 | GTGCTCCCGTCACCGTTTCTGGCACCAGG |
| 12462_L1 | GTGCGGAAAAGCGATCAAGTGCTGCAGAAGGGAT |
| 12463_L1 | CACCGACATCGATGTCCTCCCCAT |
| 12464_L1 | CACCGATGAAGCACTGTGGGTACGA |
| 12466_L1 | CACCGCCCTTCAGCTAAAATAAAGG |
| 12454_L1 | AAAACAGACTGAGCACGCTTTGATGGCAGA |
| 12455_L1 | AAAATCCGAGCAGAAGAAGAAGGCTCCCATCAC |
| 12456_L1 | AAAAATTTTCAGGAGGAAGCATGGATGGCTTCAGACA |
| 12457_L1 | AAAAATTTTCAGGAGGAAGCATGGCTTCAGACA |
| 12460_L1 | AAAACCTGGTGCCAGAAACGGTGACGGGA |
| 12462_L1 | AAAAATCCCTTCTGCAGCACTTGATCGCTTTTCC |
| 12463_L1 | AAACATGGGGAGGACATCGATGTC |
| 12464_L1 | AAACTCGTACCCACAGTGCTTCATC |
| 12466_L1 | AAACCCTTTATTTTAGCTGAAGGGC |
| 12474_peg1 | GTGCAGACTTCTCCACAGGAGTCAGGTGCAC |
| 12474-HBB-spacer_L1 | CACCGCATGGTGCACCTGACTCCTG |
| 12475_L1 | CACCGTACCTGAACCGTATATCCTA |
| 12474_peg2 | AAAAGTGCACCTGACTCCTGTGGAGAAGTCT |
| 12474-HBB-spacer_L2 | AAACCAGGAGTCAGGTGCACCATGC |
| 12475-HEXA-spacer_L2 | AAACTAGGATATACGGTTCAGGTAC |
| 12475_peg1 | GTGCAGTCAGGGCCATAGGATAGATATACGGTTC |
| 12476-PRNP-spacer_L1 | CACCGCAGTGGTGGGGGGCCTTGG |
| 12476_peg1 | GTGCATGTAGACGCCAAGGCCCCCCACC |
| 12479_L1 | CACCGCCTTGATACCAACCTGCCCA |
| 12480_L1 | CACCGCTTTCACCTTCAAATGCCA |
| 12481_L1 | CACCGCATGTTTTCACGATAGTAA |
| 12475_peg2 | AAAAGAACCGTATATCTATCCTATGGCCCTGACT |
| 12476-PRNP-spacer_L2 | AAACCCAAGGCCCCCCACCACTGC |
| 12476_peg2 | AAAAGGTGGGGGGCCTTGGCGTCTACAT |
| 12479_L2 | AAACTGGGCAGGTTGGTATCAAGGC |
| 12480_L2 | AAACTGGCATTTGAAGGTGAAAGC |
| 12481_L2 | AAACTTACTATCGTGAAAACATGC |
|  |  |
| **Primers for genomic PCR** | |
| HEK3_for-i5 | TCGTCGGCAGCGTCAGATGTGTATAAGAGACAGATGTGGGCTGCCTAGAAAGG |
| HEK3_rev-i7 | GTCTCGTGGGCTCGGAGATGTGTATAAGAGACAGCCCAGCCAAACTTGTCAACC |
| RNF2_for-i5 | TCGTCGGCAGCGTCAGATGTGTATAAGAGACAGACGTCTCATATGCCCCTTGG |
| RNF2_rev-i7 | GTCTCGTGGGCTCGGAGATGTGTATAAGAGACAGACGTAGGAATTTTGGTGGGACA |
| FANCF2-for-i5 | TCGTCGGCAGCGTCAGATGTGTATAAGAGACAGGGTGCTGACGTAGGTAGTGC |
| FANCF2-rev-i7 | GTCTCGTGGGCTCGGAGATGTGTATAAGAGACAGACACGGATAAAGACGCTGGG |
| EMX1_for-i5 | TCGTCGGCAGCGTCAGATGTGTATAAGAGACAGCAGCTCAGCCTGAGTGTTGA |
| EMX1_rev-i7 | GTCTCGTGGGCTCGGAGATGTGTATAAGAGACAGCTCGTGGGTTTGTGGTTGC |
| HEK4_for-i5 | TCGTCGGCAGCGTCAGATGTGTATAAGAGACAGGAACCCAGGTAGCCAGAGAC |
| HEK4_rev-i7 | GTCTCGTGGGCTCGGAGATGTGTATAAGAGACAGTCCTTTCAACCCGAACGGAG |
| HEK3_OT1-fwd-i7 | GTCTCGTGGGCTCGGAGATGTGTATAAGAGACAGTCCCCTGTTGACCTGGAGAA |
| HEK3_OT1-rev-i5 | TCGTCGGCAGCGTCAGATGTGTATAAGAGACAGCACTGTACTTGCCCTGACCA |
| HEK3_OT2-fwd-i5 | TCGTCGGCAGCGTCAGATGTGTATAAGAGACAGTTGGTGTTGACAGGGAGCAA |
| HEK3_OT2-rev-i7 | GTCTCGTGGGCTCGGAGATGTGTATAAGAGACAGCTGAGATGTGGGCAGAAGGG |
| HEK3_OT3-fwd-i5 | TCGTCGGCAGCGTCAGATGTGTATAAGAGACAGTGAGAGGGAACAGAAGGGCT |
| HEK3_OT3-rev-i7 | GTCTCGTGGGCTCGGAGATGTGTATAAGAGACAGGTCCAAAGGCCCAAGAACCT |
| HEK3_OT4-fwd-i5 | TCGTCGGCAGCGTCAGATGTGTATAAGAGACAGTCCTAGCACTTTGGAAGGTCG |
| HEK3_OT4-rev-i7 | GTCTCGTGGGCTCGGAGATGTGTATAAGAGACAGGCTCATCTTAATCTGCTCAGCC |
| HEK4_OT1-fwd-i7 | GTCTCGTGGGCTCGGAGATGTGTATAAGAGACAGGGCATGGCTTCTGAGACTCA |
| HEK4_OT1-rev-i5 | TCGTCGGCAGCGTCAGATGTGTATAAGAGACAGGTCTCCCTTGCACTCCCTGTCTTT |
| HEK4_OT2-fwd-i5 | TCGTCGGCAGCGTCAGATGTGTATAAGAGACAGTTTGGCAATGGAGGCATTGG |
| HEK4_OT2-rev-i7 | GTCTCGTGGGCTCGGAGATGTGTATAAGAGACAGGAAGAGGCTGCCCATGAGAG |
| HEK4_OT3-fwd-i7 | GTCTCGTGGGCTCGGAGATGTGTATAAGAGACAGGGTCTGAGGCTCGAATCCTG |
| HEK4_OT3-rev-i5 | TCGTCGGCAGCGTCAGATGTGTATAAGAGACAGCTGTGGCCTCCATATCCCTG |
| HEK4_OT4-fwd-i7 | GTCTCGTGGGCTCGGAGATGTGTATAAGAGACAGTTTCCACCAGAACTCAGCCC |
| HEK4_OT4-rev-i5 | TCGTCGGCAGCGTCAGATGTGTATAAGAGACAGCCTCGGTTCCTCCACAACAC |
| EMX1_OT1-fwd-i7 | GTCTCGTGGGCTCGGAGATGTGTATAAGAGACAGGTGGGGAGATTTGCATCTGTGGAGG |
| EMX1_OT1-rev-i5 | TCGTCGGCAGCGTCAGATGTGTATAAGAGACAGGCTTTTATACCATCTTGGGGTTACAG |
| EMX1_OT2-fwd-i5 | TCGTCGGCAGCGTCAGATGTGTATAAGAGACAGCAATGTGCTTCAACCCATCACGGC |
| EMX1_OT2-rev-i7 | GTCTCGTGGGCTCGGAGATGTGTATAAGAGACAGCCATGAATTTGTGATGGATGCAGTCTG |
| EMX1_OT3-fwd-i5 | TCGTCGGCAGCGTCAGATGTGTATAAGAGACAGGAGAAGGAGGTGCAGGAGCTAGAC |
| EMX1_OT3-rev-i7 | GTCTCGTGGGCTCGGAGATGTGTATAAGAGACAGCATCCCGACCTTCATCCCTCCTGG |
| FANCF_OT1-fwd-i5 | TCGTCGGCAGCGTCAGATGTGTATAAGAGACAGGCGGGCAGTGGCGTCTTAGTCG |
| FANCF_OT1-rev-i7 | GTCTCGTGGGCTCGGAGATGTGTATAAGAGACAGCCCTGGGTTTGGTTGGCTGCTC |
| FANCF_OT2-fwd-i5 | TCGTCGGCAGCGTCAGATGTGTATAAGAGACAGCTCCTTGCCGCCCAGCCGGTC |
| FANCF_OT2-rev-i7 | GTCTCGTGGGCTCGGAGATGTGTATAAGAGACAGCACTGGGGAAGAGGCGAGGACAC |
| FANCF_OT3-fwd-i5 | TCGTCGGCAGCGTCAGATGTGTATAAGAGACAGCCAGTGTTTCCCATCCCCAACAC |
| FANCF_OT3-rev-i7 | GTCTCGTGGGCTCGGAGATGTGTATAAGAGACAGGAATGGATCCCCCCCTAGAGCTC |
| FANCF_OT4-fwd-i5 | TCGTCGGCAGCGTCAGATGTGTATAAGAGACAGCAGGCCCACAGGTCCTTCTGGA |
| FANCF_OT4-rev-i7 | GTCTCGTGGGCTCGGAGATGTGTATAAGAGACAGCCACACGGAAGGCTGACCACG |
| HBB_ON-fwd-i7 | GTCTCGTGGGCTCGGAGATGTGTATAAGAGACAGAGGGTTGGCCAATCTACTCCC |
| HBB_ON-rev-i5 | TCGTCGGCAGCGTCAGATGTGTATAAGAGACAGGTCTTCTCTGTCTCCACATGCC |
| PRNP_ON-fwd-i5 | TCGTCGGCAGCGTCAGATGTGTATAAGAGACAGGTCAGTGGAACAAGCCGAGT |
| PRNP_ON-rev-i7 | GTCTCGTGGGCTCGGAGATGTGTATAAGAGACAGACTTGGTTGGGGTAACGGTG |
| HEXA_ON-fwd-i7 | GTCTCGTGGGCTCGGAGATGTGTATAAGAGACAGCATACAGGTGTGGCGAGAGG |
| HEXA_ON-rev-i5 | TCGTCGGCAGCGTCAGATGTGTATAAGAGACAGCCAGCCTCCTTTGGTTAGCA |
| DNMT_ON-fwd-i5 | TCGTCGGCAGCGTCAGATGTGTATAAGAGACAGCACAACAGCTTCATGTCAGCC |
| DNMT_ON-rev-i7 | GTCTCGTGGGCTCGGAGATGTGTATAAGAGACAGACGTTAATGTTTCCTGATGGTCC |
| RUNX1-rev-i5 | TCGTCGGCAGCGTCAGATGTGTATAAGAGACAGGGGTGAGGCTGAAACAGTGACC |
| RUNX1-fwd-i7 | GTCTCGTGGGCTCGGAGATGTGTATAAGAGACAGGGGAACTGGCAGGCACCGAGG |
