# Supplementary material for: PEAR, a flexible fluorescent reporter for the identification and enrichment of successfully prime edited cells
Source: eLife. 2022 Feb 23;11:e69504. doi: 10.7554/eLife.69504 (PMC8865850; doi:10.7554/eLife.69504)
Supplement: Supplementary file 4. — This file contains the necessary information to identify the NGS samples used in this study. [file elife-69504-supp4.docx]

**Supplementary File 4 – Sample indexing for NGS**

| **Sample name** | **Cell line** | **Enrichment** | **Parallel experiment** | **Amplicon** | **i5 index** | **i7 index** | **NGS date** |
| --- | --- | --- | --- | --- | --- | --- | --- |
| 1 | K562 | no enrichment | 1 | EMX1 | S513 | N712 | 2021.08.04 |
| 2 | K562 | transfection enrichment | 1 | EMX1 | S513 | N714 | 2021.08.04 |
| 3 | K562 | PEAR enrichment | 1 | EMX1 | S513 | N715 | 2021.08.04 |
| 4 | K562 | no enrichment | 2 | EMX1 | S515 | N701 | 2021.08.04 |
| 5 | K562 | transfection enrichment | 2 | EMX1 | S515 | N702 | 2021.08.04 |
| 6 | K562 | PEAR enrichment | 2 | EMX1 | S515 | N703 | 2021.08.04 |
| 7 | K562 | no enrichment | 3 | EMX1 | S515 | N704 | 2021.08.04 |
| 8 | K562 | transfection enrichment | 3 | EMX1 | S515 | N705 | 2021.08.04 |
| 9 | K562 | PEAR enrichment | 3 | EMX1 | S515 | N706 | 2021.08.04 |
| 10 | K562 | untransfected cells | 1 | EMX1 | S515 | N712 | 2021.08.04 |
| 11 | K562 | untransfected cells | 2 | EMX1 | S515 | N714 | 2021.08.04 |
| 12 | K562 | untransfected cells | 3 | EMX1 | S515 | N715 | 2021.08.04 |
| 13 | K562 | no enrichment | 1 | RNF2 | S513 | N712 | 2021.08.04 |
| 14 | K562 | transfection enrichment | 1 | RNF2 | S513 | N714 | 2021.08.04 |
| 15 | K562 | PEAR enrichment | 1 | RNF2 | S513 | N715 | 2021.08.04 |
| 16 | K562 | no enrichment | 2 | RNF2 | S515 | N701 | 2021.08.04 |
| 17 | K562 | transfection enrichment | 2 | RNF2 | S515 | N702 | 2021.08.04 |
| 18 | K562 | PEAR enrichment | 2 | RNF2 | S515 | N703 | 2021.08.04 |
| 19 | K562 | no enrichment | 3 | RNF2 | S515 | N704 | 2021.08.04 |
| 20 | K562 | transfection enrichment | 3 | RNF2 | S515 | N705 | 2021.08.04 |
| 21 | K562 | PEAR enrichment | 3 | RNF2 | S515 | N706 | 2021.08.04 |
| 22 | K562 | untransfected cells | 1 | RNF2 | S515 | N712 | 2021.08.04 |
| 23 | K562 | untransfected cells | 2 | RNF2 | S515 | N714 | 2021.08.04 |
| 24 | K562 | untransfected cells | 3 | RNF2 | S515 | N715 | 2021.08.04 |
| 25 | K562 | no enrichment | 1 | FANCF | S513 | N712 | 2021.08.04 |
| 26 | K562 | transfection enrichment | 1 | FANCF | S513 | N714 | 2021.08.04 |
| 27 | K562 | PEAR enrichment | 1 | FANCF | S513 | N715 | 2021.08.04 |
| 28 | K562 | no enrichment | 2 | FANCF | S515 | N701 | 2021.08.04 |
| 29 | K562 | transfection enrichment | 2 | FANCF | S515 | N702 | 2021.08.04 |
| 30 | K562 | PEAR enrichment | 2 | FANCF | S515 | N703 | 2021.08.04 |
| 31 | K562 | no enrichment | 3 | FANCF | S515 | N704 | 2021.08.04 |
| 32 | K562 | transfection enrichment | 3 | FANCF | S515 | N705 | 2021.08.04 |
| 33 | K562 | PEAR enrichment | 3 | FANCF | S515 | N706 | 2021.08.04 |
| 34 | K562 | untransfected cells | 1 | FANCF | S515 | N712 | 2021.08.04 |
| 35 | K562 | untransfected cells | 2 | FANCF | S515 | N714 | 2021.08.04 |
| 36 | K562 | untransfected cells | 3 | FANCF | S515 | N715 | 2021.08.04 |
| 37 | K562 | no enrichment | 1 | HEK3 | S513 | N712 | 2021.08.04 |
| 38 | K562 | transfection enrichment | 1 | HEK3 | S513 | N714 | 2021.08.04 |
| 39 | K562 | PEAR enrichment | 1 | HEK3 | S513 | N715 | 2021.08.04 |
| 40 | K562 | no enrichment | 2 | HEK3 | S515 | N701 | 2021.08.04 |
| 41 | K562 | transfection enrichment | 2 | HEK3 | S515 | N702 | 2021.08.04 |
| 42 | K562 | PEAR enrichment | 2 | HEK3 | S515 | N703 | 2021.08.04 |
| 43 | K562 | no enrichment | 3 | HEK3 | S515 | N704 | 2021.08.04 |
| 44 | K562 | transfection enrichment | 3 | HEK3 | S515 | N705 | 2021.08.04 |
| 45 | K562 | PEAR enrichment | 3 | HEK3 | S515 | N706 | 2021.08.04 |
| 46 | K562 | untransfected cells | 1 | HEK3 | S515 | N712 | 2021.08.04 |
| 47 | K562 | untransfected cells | 2 | HEK3 | S515 | N714 | 2021.08.04 |
| 48 | K562 | untransfected cells | 3 | HEK3 | S515 | N715 | 2021.08.04 |
| 49 | HEK239T | no enrichment | 1 | EMX1-OT1 | S513 | N701 | 2021.09.06 |
| 50 | HEK239T | transfection enrichment | 1 | EMX1-OT1 | S513 | N702 | 2021.09.06 |
| 51 | HEK239T | PEAR enrichment | 1 | EMX1-OT1 | S513 | N703 | 2021.09.06 |
| 52 | HEK239T | no enrichment | 2 | EMX1-OT1 | S513 | N704 | 2021.09.06 |
| 53 | HEK239T | transfection enrichment | 2 | EMX1-OT1 | S513 | N705 | 2021.09.06 |
| 54 | HEK239T | PEAR enrichment | 2 | EMX1-OT1 | S513 | N706 | 2021.09.06 |
| 55 | HEK239T | no enrichment | 3 | EMX1-OT1 | S513 | N707 | 2021.09.06 |
| 56 | HEK239T | transfection enrichment | 3 | EMX1-OT1 | S513 | N710 | 2021.09.06 |
| 57 | HEK239T | PEAR enrichment | 3 | EMX1-OT1 | S513 | N711 | 2021.09.06 |
| 58 | HEK239T | untransfected cells | 1 | EMX1-OT1 | S513 | N712 | 2021.09.06 |
| 59 | HEK239T | untransfected cells | 2 | EMX1-OT1 | S513 | N714 | 2021.09.06 |
| 60 | HEK239T | untransfected cells | 3 | EMX1-OT1 | S513 | N715 | 2021.09.06 |
| 61 | HEK239T | no enrichment | 1 | EMX1-OT2 | S513 | N701 | 2021.09.06 |
| 62 | HEK239T | transfection enrichment | 1 | EMX1-OT2 | S513 | N702 | 2021.09.06 |
| 63 | HEK239T | PEAR enrichment | 1 | EMX1-OT2 | S513 | N703 | 2021.09.06 |
| 64 | HEK239T | no enrichment | 2 | EMX1-OT2 | S513 | N704 | 2021.09.06 |
| 65 | HEK239T | transfection enrichment | 2 | EMX1-OT2 | S513 | N705 | 2021.09.06 |
| 66 | HEK239T | PEAR enrichment | 2 | EMX1-OT2 | S513 | N706 | 2021.09.06 |
| 67 | HEK239T | no enrichment | 3 | EMX1-OT2 | S513 | N707 | 2021.09.06 |
| 68 | HEK239T | transfection enrichment | 3 | EMX1-OT2 | S513 | N710 | 2021.09.06 |
| 69 | HEK239T | PEAR enrichment | 3 | EMX1-OT2 | S513 | N711 | 2021.09.06 |
| 70 | HEK239T | untransfected cells | 1 | EMX1-OT2 | S513 | N712 | 2021.09.06 |
| 71 | HEK239T | untransfected cells | 2 | EMX1-OT2 | S513 | N714 | 2021.09.06 |
| 72 | HEK239T | untransfected cells | 3 | EMX1-OT2 | S513 | N715 | 2021.09.06 |
| 73 | HEK239T | no enrichment | 1 | RNF2 | S513 | N701 | 2021.09.06 |
| 74 | HEK239T | transfection enrichment | 1 | RNF2 | S513 | N702 | 2021.09.06 |
| 75 | HEK239T | PEAR enrichment | 1 | RNF2 | S513 | N703 | 2021.09.06 |
| 76 | HEK239T | no enrichment | 2 | RNF2 | S513 | N704 | 2021.09.06 |
| 77 | HEK239T | transfection enrichment | 2 | RNF2 | S513 | N705 | 2021.09.06 |
| 78 | HEK239T | PEAR enrichment | 2 | RNF2 | S513 | N706 | 2021.09.06 |
| 79 | HEK239T | no enrichment | 3 | RNF2 | S513 | N707 | 2021.09.06 |
| 80 | HEK239T | transfection enrichment | 3 | RNF2 | S513 | N710 | 2021.09.06 |
| 81 | HEK239T | PEAR enrichment | 3 | RNF2 | S513 | N711 | 2021.09.06 |
| 82 | HEK239T | untransfected cells | 1 | RNF2 | S516 | N701 | 2021.09.06 |
| 83 | HEK239T | untransfected cells | 2 | RNF2 | S516 | N702 | 2021.09.06 |
| 84 | HEK239T | untransfected cells | 3 | RNF2 | S516 | N703 | 2021.09.06 |
| 85 | HEK239T | no enrichment | 1 | HEK4-OT2 | S513 | N701 | 2021.09.06 |
| 86 | HEK239T | transfection enrichment | 1 | HEK4-OT2 | S513 | N702 | 2021.09.06 |
| 87 | HEK239T | PEAR enrichment | 1 | HEK4-OT2 | S513 | N703 | 2021.09.06 |
| 88 | HEK239T | no enrichment | 2 | HEK4-OT2 | S513 | N704 | 2021.09.06 |
| 89 | HEK239T | transfection enrichment | 2 | HEK4-OT2 | S513 | N705 | 2021.09.06 |
| 90 | HEK239T | PEAR enrichment | 2 | HEK4-OT2 | S513 | N706 | 2021.09.06 |
| 91 | HEK239T | no enrichment | 3 | HEK4-OT2 | S513 | N707 | 2021.09.06 |
| 92 | HEK239T | transfection enrichment | 3 | HEK4-OT2 | S513 | N710 | 2021.09.06 |
| 93 | HEK239T | PEAR enrichment | 3 | HEK4-OT2 | S513 | N711 | 2021.09.06 |
| 94 | HEK239T | untransfected cells | 1 | HEK4-OT2 | S513 | N712 | 2021.09.06 |
| 95 | HEK239T | untransfected cells | 2 | HEK4-OT2 | S513 | N714 | 2021.09.06 |
| 96 | HEK239T | untransfected cells | 3 | HEK4-OT2 | S513 | N715 | 2021.09.06 |
| 97 | HEK239T | no enrichment | 1 | EMX1 | S515 | N701 | 2021.09.06 |
| 98 | HEK239T | transfection enrichment | 1 | EMX1 | S515 | N702 | 2021.09.06 |
| 99 | HEK239T | PEAR enrichment | 1 | EMX1 | S515 | N703 | 2021.09.06 |
| 100 | HEK239T | no enrichment | 2 | EMX1 | S515 | N704 | 2021.09.06 |
| 101 | HEK239T | transfection enrichment | 2 | EMX1 | S515 | N705 | 2021.09.06 |
| 102 | HEK239T | PEAR enrichment | 2 | EMX1 | S515 | N706 | 2021.09.06 |
| 103 | HEK239T | no enrichment | 3 | EMX1 | S515 | N707 | 2021.09.06 |
| 104 | HEK239T | transfection enrichment | 3 | EMX1 | S515 | N710 | 2021.09.06 |
| 105 | HEK239T | PEAR enrichment | 3 | EMX1 | S515 | N711 | 2021.09.06 |
| 106 | HEK239T | untransfected cells | 1 | EMX1 | S518 | N701 | 2021.09.06 |
| 107 | HEK239T | untransfected cells | 2 | EMX1 | S518 | N702 | 2021.09.06 |
| 108 | HEK239T | untransfected cells | 3 | EMX1 | S518 | N703 | 2021.09.06 |
| 109 | HEK239T | no enrichment | 1 | HEK3-OT1 | S515 | N701 | 2021.09.06 |
| 110 | HEK239T | transfection enrichment | 1 | HEK3-OT1 | S515 | N702 | 2021.09.06 |
| 111 | HEK239T | PEAR enrichment | 1 | HEK3-OT1 | S515 | N703 | 2021.09.06 |
| 112 | HEK239T | no enrichment | 2 | HEK3-OT1 | S515 | N704 | 2021.09.06 |
| 113 | HEK239T | transfection enrichment | 2 | HEK3-OT1 | S515 | N705 | 2021.09.06 |
| 114 | HEK239T | PEAR enrichment | 2 | HEK3-OT1 | S515 | N706 | 2021.09.06 |
| 115 | HEK239T | no enrichment | 3 | HEK3-OT1 | S515 | N707 | 2021.09.06 |
| 116 | HEK239T | transfection enrichment | 3 | HEK3-OT1 | S515 | N710 | 2021.09.06 |
| 117 | HEK239T | PEAR enrichment | 3 | HEK3-OT1 | S515 | N711 | 2021.09.06 |
| 118 | HEK239T | untransfected cells | 1 | HEK3-OT1 | S515 | N712 | 2021.09.06 |
| 119 | HEK239T | untransfected cells | 2 | HEK3-OT1 | S515 | N714 | 2021.09.06 |
| 120 | HEK239T | untransfected cells | 3 | HEK3-OT1 | S515 | N715 | 2021.09.06 |
| 121 | HEK239T | no enrichment | 1 | FANCF | S518 | N711 | 2021.09.06 |
| 122 | HEK239T | transfection enrichment | 1 | FANCF | S515 | N701 | 2021.09.06 |
| 123 | HEK239T | PEAR enrichment | 1 | FANCF | S515 | N702 | 2021.09.06 |
| 124 | HEK239T | no enrichment | 2 | FANCF | S515 | N703 | 2021.09.06 |
| 125 | HEK239T | transfection enrichment | 2 | FANCF | S515 | N704 | 2021.09.06 |
| 126 | HEK239T | PEAR enrichment | 2 | FANCF | S515 | N705 | 2021.09.06 |
| 127 | HEK239T | no enrichment | 3 | FANCF | S515 | N706 | 2021.09.06 |
| 128 | HEK239T | transfection enrichment | 3 | FANCF | S515 | N707 | 2021.09.06 |
| 129 | HEK239T | PEAR enrichment | 3 | FANCF | S515 | N710 | 2021.09.06 |
| 130 | HEK239T | untransfected cells | 1 | FANCF | S516 | N715 | 2021.09.06 |
| 131 | HEK239T | untransfected cells | 2 | FANCF | S518 | N701 | 2021.09.06 |
| 132 | HEK239T | untransfected cells | 3 | FANCF | S518 | N702 | 2021.09.06 |
| 133 | HEK239T | no enrichment | 1 | HEK3-OT2 | S515 | N701 | 2021.09.06 |
| 134 | HEK239T | transfection enrichment | 1 | HEK3-OT2 | S515 | N702 | 2021.09.06 |
| 135 | HEK239T | PEAR enrichment | 1 | HEK3-OT2 | S515 | N703 | 2021.09.06 |
| 136 | HEK239T | no enrichment | 2 | HEK3-OT2 | S515 | N704 | 2021.09.06 |
| 137 | HEK239T | transfection enrichment | 2 | HEK3-OT2 | S515 | N705 | 2021.09.06 |
| 138 | HEK239T | PEAR enrichment | 2 | HEK3-OT2 | S515 | N706 | 2021.09.06 |
| 139 | HEK239T | no enrichment | 3 | HEK3-OT2 | S515 | N707 | 2021.09.06 |
| 140 | HEK239T | transfection enrichment | 3 | HEK3-OT2 | S515 | N710 | 2021.09.06 |
| 141 | HEK239T | PEAR enrichment | 3 | HEK3-OT2 | S515 | N711 | 2021.09.06 |
| 142 | HEK239T | untransfected cells | 1 | HEK3-OT2 | S515 | N712 | 2021.09.06 |
| 143 | HEK239T | untransfected cells | 2 | HEK3-OT2 | S515 | N714 | 2021.09.06 |
| 144 | HEK239T | untransfected cells | 3 | HEK3-OT2 | S515 | N715 | 2021.09.06 |
| 145 | HEK239T | no enrichment | 1 | HEK3 | S516 | N701 | 2021.09.06 |
| 146 | HEK239T | transfection enrichment | 1 | HEK3 | S516 | N702 | 2021.09.06 |
| 147 | HEK239T | PEAR enrichment | 1 | HEK3 | S516 | N703 | 2021.09.06 |
| 148 | HEK239T | no enrichment | 2 | HEK3 | S516 | N704 | 2021.09.06 |
| 149 | HEK239T | transfection enrichment | 2 | HEK3 | S516 | N705 | 2021.09.06 |
| 150 | HEK239T | PEAR enrichment | 2 | HEK3 | S516 | N706 | 2021.09.06 |
| 151 | HEK239T | no enrichment | 3 | HEK3 | S516 | N707 | 2021.09.06 |
| 152 | HEK239T | transfection enrichment | 3 | HEK3 | S516 | N710 | 2021.09.06 |
| 153 | HEK239T | PEAR enrichment | 3 | HEK3 | S516 | N711 | 2021.09.06 |
| 154 | HEK239T | untransfected cells | 1 | HEK3 | S518 | N715 | 2021.09.06 |
| 155 | HEK239T | untransfected cells | 2 | HEK3 | S520 | N701 | 2021.09.06 |
| 156 | HEK239T | untransfected cells | 3 | HEK3 | S520 | N702 | 2021.09.06 |
| 157 | HEK239T | no enrichment | 1 | HEK3-OT3 | S518 | N701 | 2021.09.06 |
| 158 | HEK239T | transfection enrichment | 1 | HEK3-OT3 | S518 | N702 | 2021.09.06 |
| 159 | HEK239T | PEAR enrichment | 1 | HEK3-OT3 | S518 | N703 | 2021.09.06 |
| 160 | HEK239T | no enrichment | 2 | HEK3-OT3 | S518 | N704 | 2021.09.06 |
| 161 | HEK239T | transfection enrichment | 2 | HEK3-OT3 | S518 | N705 | 2021.09.06 |
| 162 | HEK239T | PEAR enrichment | 2 | HEK3-OT3 | S518 | N706 | 2021.09.06 |
| 163 | HEK239T | no enrichment | 3 | HEK3-OT3 | S518 | N707 | 2021.09.06 |
| 164 | HEK239T | transfection enrichment | 3 | HEK3-OT3 | S518 | N710 | 2021.09.06 |
| 165 | HEK239T | PEAR enrichment | 3 | HEK3-OT3 | S518 | N711 | 2021.09.06 |
| 166 | HEK239T | untransfected cells | 1 | HEK3-OT3 | S518 | N712 | 2021.09.06 |
| 167 | HEK239T | untransfected cells | 2 | HEK3-OT3 | S518 | N714 | 2021.09.06 |
| 168 | HEK239T | untransfected cells | 3 | HEK3-OT3 | S518 | N715 | 2021.09.06 |
| 169 | HEK239T | no enrichment | 1 | HEK3-OT4 | S518 | N701 | 2021.09.06 |
| 170 | HEK239T | transfection enrichment | 1 | HEK3-OT4 | S518 | N702 | 2021.09.06 |
| 171 | HEK239T | PEAR enrichment | 1 | HEK3-OT4 | S518 | N703 | 2021.09.06 |
| 172 | HEK239T | no enrichment | 2 | HEK3-OT4 | S518 | N704 | 2021.09.06 |
| 173 | HEK239T | transfection enrichment | 2 | HEK3-OT4 | S518 | N705 | 2021.09.06 |
| 174 | HEK239T | PEAR enrichment | 2 | HEK3-OT4 | S518 | N706 | 2021.09.06 |
| 175 | HEK239T | no enrichment | 3 | HEK3-OT4 | S518 | N707 | 2021.09.06 |
| 176 | HEK239T | transfection enrichment | 3 | HEK3-OT4 | S518 | N710 | 2021.09.06 |
| 177 | HEK239T | PEAR enrichment | 3 | HEK3-OT4 | S518 | N711 | 2021.09.06 |
| 178 | HEK239T | untransfected cells | 1 | HEK3-OT4 | S518 | N712 | 2021.09.06 |
| 179 | HEK239T | untransfected cells | 2 | HEK3-OT4 | S518 | N714 | 2021.09.06 |
| 180 | HEK239T | untransfected cells | 3 | HEK3-OT4 | S518 | N715 | 2021.09.06 |
| 181 | HEK239T | no enrichment | 1 | HEK4 | S520 | N701 | 2021.09.06 |
| 182 | HEK239T | transfection enrichment | 1 | HEK4 | S520 | N702 | 2021.09.06 |
| 183 | HEK239T | PEAR enrichment | 1 | HEK4 | S520 | N703 | 2021.09.06 |
| 184 | HEK239T | no enrichment | 2 | HEK4 | S520 | N704 | 2021.09.06 |
| 185 | HEK239T | transfection enrichment | 2 | HEK4 | S520 | N705 | 2021.09.06 |
| 186 | HEK239T | PEAR enrichment | 2 | HEK4 | S520 | N706 | 2021.09.06 |
| 187 | HEK239T | no enrichment | 3 | HEK4 | S520 | N707 | 2021.09.06 |
| 188 | HEK239T | transfection enrichment | 3 | HEK4 | S520 | N710 | 2021.09.06 |
| 189 | HEK239T | PEAR enrichment | 3 | HEK4 | S520 | N711 | 2021.09.06 |
| 190 | HEK239T | untransfected cells | 1 | HEK4 | S520 | N712 | 2021.09.06 |
| 191 | HEK239T | untransfected cells | 2 | HEK4 | S520 | N714 | 2021.09.06 |
| 192 | HEK239T | untransfected cells | 3 | HEK4 | S520 | N715 | 2021.09.06 |
| 193 | HEK239T | no enrichment | 1 | HEK4-OT1 | S520 | N701 | 2021.09.06 |
| 194 | HEK239T | transfection enrichment | 1 | HEK4-OT1 | S520 | N702 | 2021.09.06 |
| 195 | HEK239T | PEAR enrichment | 1 | HEK4-OT1 | S520 | N703 | 2021.09.06 |
| 196 | HEK239T | no enrichment | 2 | HEK4-OT1 | S520 | N704 | 2021.09.06 |
| 197 | HEK239T | transfection enrichment | 2 | HEK4-OT1 | S520 | N705 | 2021.09.06 |
| 198 | HEK239T | PEAR enrichment | 2 | HEK4-OT1 | S520 | N706 | 2021.09.06 |
| 199 | HEK239T | no enrichment | 3 | HEK4-OT1 | S520 | N707 | 2021.09.06 |
| 200 | HEK239T | transfection enrichment | 3 | HEK4-OT1 | S520 | N710 | 2021.09.06 |
| 201 | HEK239T | PEAR enrichment | 3 | HEK4-OT1 | S520 | N711 | 2021.09.06 |
| 202 | HEK239T | untransfected cells | 1 | HEK4-OT1 | S520 | N712 | 2021.09.06 |
| 203 | HEK239T | untransfected cells | 2 | HEK4-OT1 | S520 | N714 | 2021.09.06 |
| 204 | HEK239T | untransfected cells | 3 | HEK4-OT1 | S520 | N715 | 2021.09.06 |
| 205 | HEK239T | no enrichment | 1 | HEK4-OT4 | S520 | N701 | 2021.09.06 |
| 206 | HEK239T | transfection enrichment | 1 | HEK4-OT4 | S520 | N702 | 2021.09.06 |
| 207 | HEK239T | PEAR enrichment | 1 | HEK4-OT4 | S520 | N703 | 2021.09.06 |
| 208 | HEK239T | no enrichment | 2 | HEK4-OT4 | S520 | N704 | 2021.09.06 |
| 209 | HEK239T | transfection enrichment | 2 | HEK4-OT4 | S520 | N705 | 2021.09.06 |
| 210 | HEK239T | PEAR enrichment | 2 | HEK4-OT4 | S520 | N706 | 2021.09.06 |
| 211 | HEK239T | no enrichment | 3 | HEK4-OT4 | S520 | N707 | 2021.09.06 |
| 212 | HEK239T | transfection enrichment | 3 | HEK4-OT4 | S520 | N710 | 2021.09.06 |
| 213 | HEK239T | PEAR enrichment | 3 | HEK4-OT4 | S520 | N711 | 2021.09.06 |
| 214 | HEK239T | untransfected cells | 1 | HEK4-OT4 | S520 | N712 | 2021.09.06 |
| 215 | HEK239T | untransfected cells | 2 | HEK4-OT4 | S520 | N714 | 2021.09.06 |
| 216 | HEK239T | untransfected cells | 3 | HEK4-OT4 | S520 | N715 | 2021.09.06 |
| 217 | HEK239T | no enrichment | 1 | HEK4-OT3 | S521 | N701 | 2021.09.06 |
| 218 | HEK239T | transfection enrichment | 1 | HEK4-OT3 | S521 | N702 | 2021.09.06 |
| 219 | HEK239T | PEAR enrichment | 1 | HEK4-OT3 | S521 | N703 | 2021.09.06 |
| 220 | HEK239T | no enrichment | 2 | HEK4-OT3 | S521 | N704 | 2021.09.06 |
| 221 | HEK239T | transfection enrichment | 2 | HEK4-OT3 | S521 | N705 | 2021.09.06 |
| 222 | HEK239T | PEAR enrichment | 2 | HEK4-OT3 | S521 | N706 | 2021.09.06 |
| 223 | HEK239T | no enrichment | 3 | HEK4-OT3 | S521 | N707 | 2021.09.06 |
| 224 | HEK239T | transfection enrichment | 3 | HEK4-OT3 | S521 | N710 | 2021.09.06 |
| 225 | HEK239T | PEAR enrichment | 3 | HEK4-OT3 | S521 | N711 | 2021.09.06 |
| 226 | HEK239T | untransfected cells | 1 | HEK4-OT3 | S521 | N712 | 2021.09.06 |
| 227 | HEK239T | untransfected cells | 2 | HEK4-OT3 | S521 | N714 | 2021.09.06 |
| 228 | HEK239T | untransfected cells | 3 | HEK4-OT3 | S521 | N715 | 2021.09.06 |
| 229 | HEK239T | no enrichment | 1 | EMX1-OT3 | S521 | N701 | 2021.09.06 |
| 230 | HEK239T | transfection enrichment | 1 | EMX1-OT3 | S521 | N702 | 2021.09.06 |
| 231 | HEK239T | PEAR enrichment | 1 | EMX1-OT3 | S521 | N703 | 2021.09.06 |
| 232 | HEK239T | no enrichment | 2 | EMX1-OT3 | S521 | N704 | 2021.09.06 |
| 233 | HEK239T | transfection enrichment | 2 | EMX1-OT3 | S521 | N705 | 2021.09.06 |
| 234 | HEK239T | PEAR enrichment | 2 | EMX1-OT3 | S521 | N706 | 2021.09.06 |
| 235 | HEK239T | no enrichment | 3 | EMX1-OT3 | S521 | N707 | 2021.09.06 |
| 236 | HEK239T | transfection enrichment | 3 | EMX1-OT3 | S521 | N710 | 2021.09.06 |
| 237 | HEK239T | PEAR enrichment | 3 | EMX1-OT3 | S521 | N711 | 2021.09.06 |
| 238 | HEK239T | untransfected cells | 1 | EMX1-OT3 | S521 | N712 | 2021.09.06 |
| 239 | HEK239T | untransfected cells | 2 | EMX1-OT3 | S521 | N714 | 2021.09.06 |
| 240 | HEK239T | untransfected cells | 3 | EMX1-OT3 | S521 | N715 | 2021.09.06 |
| 241 | HEK239T | no enrichment | 1 | FANCF-OT1 | S521 | N701 | 2021.09.06 |
| 242 | HEK239T | transfection enrichment | 1 | FANCF-OT1 | S521 | N702 | 2021.09.06 |
| 243 | HEK239T | PEAR enrichment | 1 | FANCF-OT1 | S521 | N703 | 2021.09.06 |
| 244 | HEK239T | no enrichment | 2 | FANCF-OT1 | S521 | N704 | 2021.09.06 |
| 245 | HEK239T | transfection enrichment | 2 | FANCF-OT1 | S521 | N705 | 2021.09.06 |
| 246 | HEK239T | PEAR enrichment | 2 | FANCF-OT1 | S521 | N706 | 2021.09.06 |
| 247 | HEK239T | no enrichment | 3 | FANCF-OT1 | S521 | N707 | 2021.09.06 |
| 248 | HEK239T | transfection enrichment | 3 | FANCF-OT1 | S521 | N710 | 2021.09.06 |
| 249 | HEK239T | PEAR enrichment | 3 | FANCF-OT1 | S521 | N711 | 2021.09.06 |
| 250 | HEK239T | untransfected cells | 1 | FANCF-OT1 | S521 | N712 | 2021.09.06 |
| 251 | HEK239T | untransfected cells | 2 | FANCF-OT1 | S521 | N714 | 2021.09.06 |
| 252 | HEK239T | untransfected cells | 3 | FANCF-OT1 | S521 | N715 | 2021.09.06 |
| 253 | HEK239T | no enrichment | 1 | FANCF-OT2 | S522 | N701 | 2021.09.06 |
| 254 | HEK239T | transfection enrichment | 1 | FANCF-OT2 | S522 | N702 | 2021.09.06 |
| 255 | HEK239T | PEAR enrichment | 1 | FANCF-OT2 | S522 | N703 | 2021.09.06 |
| 256 | HEK239T | no enrichment | 2 | FANCF-OT2 | S522 | N704 | 2021.09.06 |
| 257 | HEK239T | transfection enrichment | 2 | FANCF-OT2 | S522 | N705 | 2021.09.06 |
| 258 | HEK239T | PEAR enrichment | 2 | FANCF-OT2 | S522 | N706 | 2021.09.06 |
| 259 | HEK239T | no enrichment | 3 | FANCF-OT2 | S522 | N707 | 2021.09.06 |
| 260 | HEK239T | transfection enrichment | 3 | FANCF-OT2 | S522 | N710 | 2021.09.06 |
| 261 | HEK239T | PEAR enrichment | 3 | FANCF-OT2 | S522 | N711 | 2021.09.06 |
| 262 | HEK239T | untransfected cells | 1 | FANCF-OT2 | S522 | N712 | 2021.09.06 |
| 263 | HEK239T | untransfected cells | 2 | FANCF-OT2 | S522 | N714 | 2021.09.06 |
| 264 | HEK239T | untransfected cells | 3 | FANCF-OT2 | S522 | N715 | 2021.09.06 |
| 265 | HEK239T | no enrichment | 1 | FANCF-OT3 | S522 | N701 | 2021.09.06 |
| 266 | HEK239T | transfection enrichment | 1 | FANCF-OT3 | S522 | N702 | 2021.09.06 |
| 267 | HEK239T | PEAR enrichment | 1 | FANCF-OT3 | S522 | N703 | 2021.09.06 |
| 268 | HEK239T | no enrichment | 2 | FANCF-OT3 | S522 | N704 | 2021.09.06 |
| 269 | HEK239T | transfection enrichment | 2 | FANCF-OT3 | S522 | N705 | 2021.09.06 |
| 270 | HEK239T | PEAR enrichment | 2 | FANCF-OT3 | S522 | N706 | 2021.09.06 |
| 271 | HEK239T | no enrichment | 3 | FANCF-OT3 | S522 | N707 | 2021.09.06 |
| 272 | HEK239T | transfection enrichment | 3 | FANCF-OT3 | S522 | N710 | 2021.09.06 |
| 273 | HEK239T | PEAR enrichment | 3 | FANCF-OT3 | S522 | N711 | 2021.09.06 |
| 274 | HEK239T | untransfected cells | 1 | FANCF-OT3 | S522 | N712 | 2021.09.06 |
| 275 | HEK239T | untransfected cells | 2 | FANCF-OT3 | S522 | N714 | 2021.09.06 |
| 276 | HEK239T | untransfected cells | 3 | FANCF-OT3 | S522 | N715 | 2021.09.06 |
| 277 | HEK239T | no enrichment | 1 | FANCF-OT4 | S522 | N701 | 2021.09.06 |
| 278 | HEK239T | transfection enrichment | 1 | FANCF-OT4 | S522 | N702 | 2021.09.06 |
| 279 | HEK239T | PEAR enrichment | 1 | FANCF-OT4 | S522 | N703 | 2021.09.06 |
| 280 | HEK239T | no enrichment | 2 | FANCF-OT4 | S522 | N704 | 2021.09.06 |
| 281 | HEK239T | transfection enrichment | 2 | FANCF-OT4 | S522 | N705 | 2021.09.06 |
| 282 | HEK239T | PEAR enrichment | 2 | FANCF-OT4 | S522 | N706 | 2021.09.06 |
| 283 | HEK239T | no enrichment | 3 | FANCF-OT4 | S522 | N707 | 2021.09.06 |
| 284 | HEK239T | transfection enrichment | 3 | FANCF-OT4 | S522 | N710 | 2021.09.06 |
| 285 | HEK239T | PEAR enrichment | 3 | FANCF-OT4 | S522 | N711 | 2021.09.06 |
| 286 | HEK239T | untransfected cells | 1 | FANCF-OT4 | S522 | N712 | 2021.09.06 |
| 287 | HEK239T | untransfected cells | 2 | FANCF-OT4 | S522 | N714 | 2021.09.06 |
| 288 | HEK239T | untransfected cells | 3 | FANCF-OT4 | S522 | N715 | 2021.09.06 |
| 289 | U2OS | no enrichment | 1 | EMX1 | S513 | N701 | 2021.09.10 |
| 290 | U2OS | transfection enrichment | 1 | EMX1 | S513 | N702 | 2021.09.10 |
| 291 | U2OS | PEAR enrichment | 1 | EMX1 | S513 | N703 | 2021.09.10 |
| 292 | U2OS | no enrichment | 2 | EMX1 | S513 | N704 | 2021.09.10 |
| 293 | U2OS | transfection enrichment | 2 | EMX1 | S513 | N705 | 2021.09.10 |
| 294 | U2OS | PEAR enrichment | 2 | EMX1 | S513 | N706 | 2021.09.10 |
| 295 | U2OS | no enrichment | 3 | EMX1 | S513 | N707 | 2021.09.10 |
| 296 | U2OS | transfection enrichment | 3 | EMX1 | S513 | N710 | 2021.09.10 |
| 297 | U2OS | PEAR enrichment | 3 | EMX1 | S513 | N711 | 2021.09.10 |
| 298 | U2OS | untransfected cells | 1 | EMX1 | S513 | N712 | 2021.09.10 |
| 299 | U2OS | untransfected cells | 2 | EMX1 | S513 | N714 | 2021.09.10 |
| 300 | U2OS | untransfected cells | 3 | EMX1 | S513 | N715 | 2021.09.10 |
| 301 | U2OS | no enrichment | 1 | RNF2 | S513 | N701 | 2021.09.10 |
| 302 | U2OS | transfection enrichment | 1 | RNF2 | S513 | N702 | 2021.09.10 |
| 303 | U2OS | PEAR enrichment | 1 | RNF2 | S513 | N703 | 2021.09.10 |
| 304 | U2OS | no enrichment | 2 | RNF2 | S513 | N704 | 2021.09.10 |
| 305 | U2OS | transfection enrichment | 2 | RNF2 | S513 | N705 | 2021.09.10 |
| 306 | U2OS | PEAR enrichment | 2 | RNF2 | S513 | N706 | 2021.09.10 |
| 307 | U2OS | no enrichment | 3 | RNF2 | S513 | N707 | 2021.09.10 |
| 308 | U2OS | transfection enrichment | 3 | RNF2 | S513 | N710 | 2021.09.10 |
| 309 | U2OS | PEAR enrichment | 3 | RNF2 | S513 | N711 | 2021.09.10 |
| 310 | U2OS | untransfected cells | 1 | RNF2 | S515 | N704 | 2021.09.10 |
| 311 | U2OS | untransfected cells | 2 | RNF2 | S515 | N705 | 2021.09.10 |
| 312 | U2OS | untransfected cells | 3 | RNF2 | S515 | N706 | 2021.09.10 |
| 313 | HUES9 | no enrichment | 1 | FANCF | S513 | N712 | 2021.09.10 |
| 314 | HUES9 | transfection enrichment | 1 | FANCF | S513 | N714 | 2021.09.10 |
| 315 | HUES9 | PEAR enrichment | 1 | FANCF | S513 | N715 | 2021.09.10 |
| 316 | HUES9 | no enrichment | 2 | FANCF | S515 | N701 | 2021.09.10 |
| 317 | HUES9 | transfection enrichment | 2 | FANCF | S515 | N702 | 2021.09.10 |
| 318 | HUES9 | PEAR enrichment | 2 | FANCF | S515 | N703 | 2021.09.10 |
| 319 | HUES9 | no enrichment | 3 | FANCF | S515 | N704 | 2021.09.10 |
| 320 | HUES9 | transfection enrichment | 3 | FANCF | S515 | N705 | 2021.09.10 |
| 321 | HUES9 | PEAR enrichment | 3 | FANCF | S515 | N706 | 2021.09.10 |
| 322 | HUES9 | untransfected cells | 1 | FANCF | S515 | N712 | 2021.09.10 |
| 323 | HUES9 | untransfected cells | 2 | FANCF | S515 | N714 | 2021.09.10 |
| 324 | HUES9 | untransfected cells | 3 | FANCF | S515 | N715 | 2021.09.10 |
| 325 | U2OS | no enrichment | 1 | HEK3 | S513 | N701 | 2021.09.10 |
| 326 | U2OS | transfection enrichment | 1 | HEK3 | S513 | N702 | 2021.09.10 |
| 327 | U2OS | PEAR enrichment | 1 | HEK3 | S513 | N703 | 2021.09.10 |
| 328 | U2OS | no enrichment | 2 | HEK3 | S513 | N704 | 2021.09.10 |
| 329 | U2OS | transfection enrichment | 2 | HEK3 | S513 | N705 | 2021.09.10 |
| 330 | U2OS | PEAR enrichment | 2 | HEK3 | S513 | N706 | 2021.09.10 |
| 331 | U2OS | no enrichment | 3 | HEK3 | S513 | N707 | 2021.09.10 |
| 332 | U2OS | transfection enrichment | 3 | HEK3 | S513 | N710 | 2021.09.10 |
| 333 | U2OS | PEAR enrichment | 3 | HEK3 | S513 | N711 | 2021.09.10 |
| 334 | U2OS | untransfected cells | 1 | HEK3 | S513 | N712 | 2021.09.10 |
| 335 | U2OS | untransfected cells | 2 | HEK3 | S513 | N714 | 2021.09.10 |
| 336 | U2OS | untransfected cells | 3 | HEK3 | S513 | N715 | 2021.09.10 |
| 337 | HEK239T | no enrichment | 1 | FANCF | S515 | N712 | 2021.09.16 |
| 338 | HEK239T | transfection enrichment | 1 | FANCF | S515 | N714 | 2021.09.16 |
| 339 | HEK239T | PEAR enrichment | 1 | FANCF | S515 | N715 | 2021.09.16 |
| 340 | HEK239T | no enrichment | 2 | FANCF | S516 | N701 | 2021.09.16 |
| 341 | HEK239T | transfection enrichment | 2 | FANCF | S516 | N702 | 2021.09.16 |
| 342 | HEK239T | PEAR enrichment | 2 | FANCF | S516 | N703 | 2021.09.16 |
| 343 | HEK239T | no enrichment | 3 | FANCF | S516 | N704 | 2021.09.16 |
| 344 | HEK239T | transfection enrichment | 3 | FANCF | S516 | N705 | 2021.09.16 |
| 345 | HEK239T | PEAR enrichment | 3 | FANCF | S516 | N706 | 2021.09.16 |
| 346 | HEK239T | untransfected cells | 1 | FANCF | S518 | N704 | 2021.09.16 |
| 347 | HEK239T | untransfected cells | 2 | FANCF | S518 | N705 | 2021.09.16 |
| 348 | HEK239T | untransfected cells | 3 | FANCF | S518 | N706 | 2021.09.16 |
| 349 | HEK239T | no enrichment | 1 | EMX1 | S515 | N701 | 2021.09.16 |
| 350 | HEK239T | transfection enrichment | 1 | EMX1 | S515 | N702 | 2021.09.16 |
| 351 | HEK239T | PEAR enrichment | 1 | EMX1 | S515 | N703 | 2021.09.16 |
| 352 | HEK239T | no enrichment | 2 | EMX1 | S515 | N704 | 2021.09.16 |
| 353 | HEK239T | transfection enrichment | 2 | EMX1 | S515 | N705 | 2021.09.16 |
| 354 | HEK239T | PEAR enrichment | 2 | EMX1 | S515 | N706 | 2021.09.16 |
| 355 | HEK239T | no enrichment | 3 | EMX1 | S515 | N707 | 2021.09.16 |
| 356 | HEK239T | transfection enrichment | 3 | EMX1 | S515 | N710 | 2021.09.16 |
| 357 | HEK239T | PEAR enrichment | 3 | EMX1 | S515 | N711 | 2021.09.16 |
| 358 | HEK239T | untransfected cells | 1 | EMX1 | S515 | N712 | 2021.09.16 |
| 359 | HEK239T | untransfected cells | 2 | EMX1 | S515 | N714 | 2021.09.16 |
| 360 | HEK239T | untransfected cells | 3 | EMX1 | S515 | N715 | 2021.09.16 |
| 361 | HEK239T | no enrichment | 1 | PRNP | S515 | N701 | 2021.09.16 |
| 362 | HEK239T | transfection enrichment | 1 | PRNP | S515 | N702 | 2021.09.16 |
| 363 | HEK239T | PEAR enrichment | 1 | PRNP | S515 | N703 | 2021.09.16 |
| 364 | HEK239T | no enrichment | 2 | PRNP | S515 | N704 | 2021.09.16 |
| 365 | HEK239T | transfection enrichment | 2 | PRNP | S515 | N705 | 2021.09.16 |
| 366 | HEK239T | PEAR enrichment | 2 | PRNP | S515 | N706 | 2021.09.16 |
| 367 | HEK239T | no enrichment | 3 | PRNP | S515 | N707 | 2021.09.16 |
| 368 | HEK239T | transfection enrichment | 3 | PRNP | S515 | N710 | 2021.09.16 |
| 369 | HEK239T | PEAR enrichment | 3 | PRNP | S515 | N711 | 2021.09.16 |
| 370 | HEK239T | untransfected cells | 1 | PRNP | S515 | N712 | 2021.09.16 |
| 371 | HEK239T | untransfected cells | 2 | PRNP | S515 | N714 | 2021.09.16 |
| 372 | HEK239T | untransfected cells | 3 | PRNP | S515 | N715 | 2021.09.16 |
| 373 | HEK239T | no enrichment | 1 | DNMT | S520 | N701 | 2021.09.16 |
| 374 | HEK239T | transfection enrichment | 1 | DNMT | S520 | N702 | 2021.09.16 |
| 375 | HEK239T | PEAR enrichment | 1 | DNMT | S520 | N703 | 2021.09.16 |
| 376 | HEK239T | no enrichment | 2 | DNMT | S520 | N704 | 2021.09.16 |
| 377 | HEK239T | transfection enrichment | 2 | DNMT | S520 | N705 | 2021.09.16 |
| 378 | HEK239T | PEAR enrichment | 2 | DNMT | S520 | N706 | 2021.09.16 |
| 379 | HEK239T | no enrichment | 3 | DNMT | S520 | N707 | 2021.09.16 |
| 380 | HEK239T | transfection enrichment | 3 | DNMT | S520 | N710 | 2021.09.16 |
| 381 | HEK239T | PEAR enrichment | 3 | DNMT | S520 | N711 | 2021.09.16 |
| 382 | HEK239T | untransfected cells | 1 | DNMT | S520 | N712 | 2021.09.16 |
| 383 | HEK239T | untransfected cells | 2 | DNMT | S520 | N714 | 2021.09.16 |
| 384 | HEK239T | untransfected cells | 3 | DNMT | S520 | N715 | 2021.09.16 |
| 385 | HEK239T | no enrichment | 1 | HEXA | S520 | N701 | 2021.09.16 |
| 386 | HEK239T | transfection enrichment | 1 | HEXA | S520 | N702 | 2021.09.16 |
| 387 | HEK239T | PEAR enrichment | 1 | HEXA | S520 | N703 | 2021.09.16 |
| 388 | HEK239T | no enrichment | 2 | HEXA | S520 | N704 | 2021.09.16 |
| 389 | HEK239T | transfection enrichment | 2 | HEXA | S520 | N705 | 2021.09.16 |
| 390 | HEK239T | PEAR enrichment | 2 | HEXA | S520 | N706 | 2021.09.16 |
| 391 | HEK239T | no enrichment | 3 | HEXA | S520 | N707 | 2021.09.16 |
| 392 | HEK239T | transfection enrichment | 3 | HEXA | S520 | N710 | 2021.09.16 |
| 393 | HEK239T | PEAR enrichment | 3 | HEXA | S520 | N711 | 2021.09.16 |
| 394 | HEK239T | untransfected cells | 1 | HEXA | S520 | N712 | 2021.09.16 |
| 395 | HEK239T | untransfected cells | 2 | HEXA | S520 | N714 | 2021.09.16 |
| 396 | HEK239T | untransfected cells | 3 | HEXA | S520 | N715 | 2021.09.16 |
| 397 | HEK239T | no enrichment | 1 | HEK3 | S518 | N701 | 2021.09.16 |
| 398 | HEK239T | transfection enrichment | 1 | HEK3 | S518 | N702 | 2021.09.16 |
| 399 | HEK239T | PEAR enrichment | 1 | HEK3 | S518 | N703 | 2021.09.16 |
| 400 | HEK239T | no enrichment | 2 | HEK3 | S518 | N704 | 2021.09.16 |
| 401 | HEK239T | transfection enrichment | 2 | HEK3 | S518 | N705 | 2021.09.16 |
| 402 | HEK239T | PEAR enrichment | 2 | HEK3 | S518 | N706 | 2021.09.16 |
| 403 | HEK239T | no enrichment | 3 | HEK3 | S518 | N707 | 2021.09.16 |
| 404 | HEK239T | transfection enrichment | 3 | HEK3 | S518 | N710 | 2021.09.16 |
| 405 | HEK239T | PEAR enrichment | 3 | HEK3 | S518 | N711 | 2021.09.16 |
| 406 | HEK239T | untransfected cells | 1 | HEK3 | S518 | N712 | 2021.09.16 |
| 407 | HEK239T | untransfected cells | 2 | HEK3 | S518 | N714 | 2021.09.16 |
| 408 | HEK239T | untransfected cells | 3 | HEK3 | S518 | N715 | 2021.09.16 |
| 409 | HEK239T | no enrichment | 1 | HBB | S518 | N701 | 2021.09.16 |
| 410 | HEK239T | transfection enrichment | 1 | HBB | S518 | N702 | 2021.09.16 |
| 411 | HEK239T | PEAR enrichment | 1 | HBB | S518 | N703 | 2021.09.16 |
| 412 | HEK239T | no enrichment | 2 | HBB | S518 | N704 | 2021.09.16 |
| 413 | HEK239T | transfection enrichment | 2 | HBB | S518 | N705 | 2021.09.16 |
| 414 | HEK239T | PEAR enrichment | 2 | HBB | S518 | N706 | 2021.09.16 |
| 415 | HEK239T | no enrichment | 3 | HBB | S518 | N707 | 2021.09.16 |
| 416 | HEK239T | transfection enrichment | 3 | HBB | S518 | N710 | 2021.09.16 |
| 417 | HEK239T | PEAR enrichment | 3 | HBB | S518 | N711 | 2021.09.16 |
| 418 | HEK239T | untransfected cells | 1 | HBB | S518 | N712 | 2021.09.16 |
| 419 | HEK239T | untransfected cells | 2 | HBB | S518 | N714 | 2021.09.16 |
| 420 | HEK239T | untransfected cells | 3 | HBB | S518 | N715 | 2021.09.16 |
| 421 | HEK239T | no enrichment | 1 | RUNX1 | S518 | N701 | 2021.09.16 |
| 422 | HEK239T | transfection enrichment | 1 | RUNX1 | S518 | N702 | 2021.09.16 |
| 423 | HEK239T | PEAR enrichment | 1 | RUNX1 | S518 | N703 | 2021.09.16 |
| 424 | HEK239T | no enrichment | 2 | RUNX1 | S518 | N704 | 2021.09.16 |
| 425 | HEK239T | transfection enrichment | 2 | RUNX1 | S518 | N705 | 2021.09.16 |
| 426 | HEK239T | PEAR enrichment | 2 | RUNX1 | S518 | N706 | 2021.09.16 |
| 427 | HEK239T | no enrichment | 3 | RUNX1 | S518 | N707 | 2021.09.16 |
| 428 | HEK239T | transfection enrichment | 3 | RUNX1 | S518 | N710 | 2021.09.16 |
| 429 | HEK239T | PEAR enrichment | 3 | RUNX1 | S518 | N711 | 2021.09.16 |
| 430 | HEK239T | no enrichment | 1 | RUNX1 | S518 | N712 | 2021.09.16 |
| 431 | HEK239T | transfection enrichment | 1 | RUNX1 | S518 | N715 | 2021.09.16 |
| 432 | HEK239T | PEAR enrichment | 1 | RUNX1 | S520 | N701 | 2021.09.16 |
| 433 | HEK239T | no enrichment | 2 | RUNX1 | S520 | N702 | 2021.09.16 |
| 434 | HEK239T | transfection enrichment | 2 | RUNX1 | S520 | N703 | 2021.09.16 |
| 435 | HEK239T | PEAR enrichment | 2 | RUNX1 | S520 | N704 | 2021.09.16 |
| 436 | HEK239T | no enrichment | 3 | RUNX1 | S520 | N705 | 2021.09.16 |
| 437 | HEK239T | transfection enrichment | 3 | RUNX1 | S520 | N706 | 2021.09.16 |
| 438 | HEK239T | PEAR enrichment | 3 | RUNX1 | S520 | N707 | 2021.09.16 |
| 439 | HEK239T | untransfected cells | 1 | RUNX1 | S520 | N710 | 2021.09.16 |
| 440 | HEK239T | untransfected cells | 2 | RUNX1 | S520 | N711 | 2021.09.16 |
| 441 | HEK239T | untransfected cells | 3 | RUNX1 | S520 | N712 | 2021.09.16 |
